# Hematological Abnormalities in COVID-19 Disease: Association With Type I Interferon Pathway Activation and Disease Outcomes

**DOI:** 10.3389/fmed.2022.850472

**Published:** 2022-03-17

**Authors:** Vasiliki E. Georgakopoulou, Panagiotis Lembessis, Charalampos Skarlis, Aikaterini Gkoufa, Nikolaos V. Sipsas, Clio P. Mavragani

**Affiliations:** ^1^Department of Infectious Diseases and COVID-19 Unit, Laiko General Hospital, School of Medicine, National and Kapodistrian University of Athens, Athens, Greece; ^2^Department of Physiology, School of Medicine, National and Kapodistrian University of Athens, Athens, Greece; ^3^Department of Pathophysiology, School of Medicine, National and Kapodistrian University of Athens, Athens, Greece; ^4^Fourth Department of Internal Medicine, School of Medicine, University Hospital Attikon, National and Kapodistrian University of Athens, Athens, Greece; ^5^Joint Academic Rheumatology Program, National and Kapodistrian University of Athens, Athens, Greece

**Keywords:** COVID-19, type I interferon, white blood cells, platelets, eosinophils, cytopenias, eosinopenia, lymphopenia

## Abstract

Increased expression of interferon (IFN)-stimulated genes (ISGs) in peripheral blood, has been previously reported in viral infections, as well as in autoimmune disorders, in association with reduced leukocyte and platelet counts. Though cytopenias are common in patients with COVID-19 disease and predict severe outcomes, the underlying mechanisms have not been fully elucidated. In the current study, we aimed to determine the prevalence of hematological abnormalities in the setting of active COVID-19 infection and to explore whether they associate with disease outcomes and activation of type I IFN pathway. One-hundred-twenty-three consecutive SARS-CoV2 infected patients were included in the study. Clinical and laboratory parameters were recorded for all study participants. In 114 patients, total RNA was extracted from whole peripheral blood and subjected to real time PCR. The relative expression of three interferon stimulated genes (ISGs; IFIT1, MX-1, and IFI44) was determined and a type I IFN score reflecting peripheral type I IFN activity was calculated. The rates of anemia, leukopenia, and thrombocytopenia were 28.5, 14.6, and 24.4%, respectively. Among leukocytopenias, eosinopenia, and lymphopenia were the most prominent abnormalities being found in 56.9 and 43.1%, respectively. Of interest, patients with either eosinopenia and/or thrombocytopenia but no other hematological abnormalities displayed significantly increased peripheral type I IFN scores compared to their counterparts with normal/high eosinophil and platelet counts. While eosinopenia along with lymphopenia were found to be associated with increased risk for intubation and severe/critical disease, such an association was not detected between other hematological abnormalities or increased type I IFN scores. In conclusion, hematological abnormalities are commonly detected among patients with COVID-19 infection in association with severe disease outcomes and activation of the type I IFN pathway.

## Introduction

Type I interferons (IFNα and IFNβ) are secreted by almost all cells in the body in response to the stimulation of cell surface and intracellular pattern recognition receptors (PRRs) by viral and microbial products. They are comprised of multiple IFN-alpha subtypes, including IFN-beta, IFN-delta, IFN-epsilon, IFN-kappa, IFN-tau, IFN-omega, and IFN-zeta, all of which have significant structural homology and bind to a common heterodimeric receptor (consisting of the IFN-alpha/beta RI and IFN-alpha/beta R2 subunits expressed on most cell types). IFNα and IFNβ are the best-defined and most broadly expressed IFNs. They act in an autocrine and paracrine manner to induce an antiviral state in both virus-infected cells and uninfected neighboring cells *via* the induction of interferon stimulated genes (ISG) (1, 2). It is well-appreciated that dysregulation of type I interferon (IFN) pathway has been implicated in the pathogenesis of autoimmune disorders such as Sjogren's Syndrome (SS) and systemic lupus erythematosus (SLE) (3, 4). In the setting of severe acute respiratory syndrome coronavirus 2 (SARS-CoV-2) infection, dysregulation of host IFN responses has been known to be associated with severe disease outcomes (5).

Several studies have demonstrated increased frequency of hematologic abnormalities in viral infections in humans (6, 7). Lymphopenia, thrombocytopenia, and eosinopenia have been also reported in patients with SARS-CoV-2 infection, in association with disease severity and mortality (8–11). However, the pathogenetic mechanisms of the hematological alterations in patients with SARS-CoV-2 infection have not been fully explored.

Given that activation of the type I IFN pathway has been previously associated with reduced platelet or leukocyte counts in viral infections and systemic autoimmune diseases such as lupus (12, 13), we aimed to explore whether increased type I IFN inducible gene expression in peripheral blood of patients with COVID-19 infection, could contribute to the observed hematological abnormalities. Moreover, the prevalence of hematological abnormalities in the setting of active COVID-19 infection and the association with disease outcomes will be also explored.

## Materials and Methods

### Study Population

Our study population is comprised of 123 consecutive newly diagnosed cases of COVID-19 infection (68 men; 47 men >50 years old, 21 men ≤ 50 years old and 55 women; 48 women >50 years old, 7 women ≤ 50 years old) who were evaluated to the outpatient department or hospitalized in COVID-19 unit of Laiko General Hospital, University of Athens School of Medicine, Greece, from October 2020 to February 2021. All patients were infected with the alpha variant (B.1.1.7).

Adults diagnosed with COVID-19 disease by a positive real-time (PCR) test in a nasopharyngeal sample were included in the study. Patients with a previous history of neoplastic disorders receiving steroids or immunosuppressive/chemotherapeutic agents or experienced a recent bacterial or parasitic infection were excluded. All patients gave written informed consent prior to participation in the study. The study conformed to the principles of the Declaration of Helsinki and was approved by the Ethics Committee of Laiko General Hospital (protocol number: 18954-14/12/2020). Patients unable to give informed consent because of impaired cognitive function were also excluded. A history of asthma was detected in five out of 123 patients (three women, two men, age range 20–58). None of them were intubated.

Detailed information about demographics, comorbidities, medication history, presenting COVID-19 related features and laboratory parameters upon initial evaluation or admission were recorded for all study participants as follows: age, sex, date of evaluation or admission, comorbidities and medications, symptoms, vital signs, date of illness, hematocrit, hemoglobin, white blood cells (WBC) count, lymphocyte, neutrophil, eosinophil and monocyte count, serum creatinine, blood urea nitrogen, aspartate transaminase (AST), alanine (ALT), gamma-glutamyl transferase (GGT), alkaline phosphatase, creatinine kinase (CK), lactate dehydrogenase (LDH), C-reactive protein (CRP), erythrocyte sedimentation rate (ESR), troponin, fibrinogen, and D-dimers. Leukopenia was defined as a reduction of WBC < 4 K/μL, neutropenia as a reduction of neutrophils <1.5 K/μL, lymphopenia as a reduction <1 K/μL, eosinopenia is as a reduction of eosinophils <0.01 K/μL, and thrombocytopenia as a reduction of platelets <150 K/μL (14–16). According to the World Health Organization (WHO), anemia is defined as hemoglobin (Hb) levels <12.0 g/dL in women and <13.0 g/dL in men.

Classification of patients was based on their disease course and according to Center for Disease Control and Prevention (CDC) clinical spectrum of SARS-CoV-2 infection, to asymptomatic, mild, moderate, severe and critical illness.^*^https://www.covid19treatmentguidelines.nih.gov/overview/clinical-spectrum.

More specifically asymptomatic cases were defined as individuals with a positive virologic test (i.e., a nucleic acid amplification test or an antigen test) for SARS-CoV-2, but who have no symptoms that are consistent with COVID-19. Mild cases were defined as individuals manifesting any of the various signs and symptoms of COVID-19 (e.g., fever, cough, sore throat, malaise, headache, muscle pain, nausea, vomiting, diarrhea, and loss of taste and smell) but who do not have shortness of breath, dyspnea, or abnormal chest imaging. As moderate cases were classified individuals with evidence of lower respiratory disease during clinical assessment or imaging and who have an oxygen saturation (SpO_2_) ≥94% on room air at sea level. As severe cases were classified individuals who have SpO_2_ <94% on room air at sea level, a ratio of arterial partial pressure of oxygen to fraction of inspired oxygen (PaO_2_/FiO_2_) <300 mm Hg, a respiratory rate >30 breaths/min, or lung infiltrates >50%. As critical cases were defined individuals who have respiratory failure, septic shock, and/or multiple organ dysfunction.

On this basis, we further divided our patient population into high (severe, critical) vs. low severity score (asymptomatic, mild, and moderate) groups.

### RNA Extraction—Quantitation of Type I IFN Score

Total RNA was extracted from whole peripheral blood derived from 114 patients with high quality RNA using the TRIzol Reagent (Invitrogen, USA) according to manufacturer's instructions and immediately stored at −80°C. The quantity and quality of RNA samples were spectrophotometrically tested (Biospec Nano, Japan).

Five hundred nanograms of total RNA was reverse transcribed using PrimeScript RT reagent Kit (Takara Japan) as per manufacturer's instructions on an Veriti cycler. All complementary DNAs were diluted 1:10 with nuclease free water (Qiagen, Germany) immediately after synthesis and stored at −20°C.

Quantitative real-time polymerase chain reaction (qRT-PCR) was implemented to quantify the expression of selected genes using the Sacace96 thermocycler and the KAPA SYBR FAST Mastermix (KAPA Biosystems, South Africa), as previously described (3). Briefly, genes preferentially induced by type I IFNs were selected, including IFN-induced protein with tetratricopeptide repeats 1 (IFIT1), interferon induced protein 44 (IFI44) and myxovirus (influenza virus) resistance 1 (MX1). Glyceraldehyde phosphate dehydrogenase (GAPDH) was used as an internal control and normalization gene (housekeeping gene). Primers specific sequences are presented in Supplementary Table 1. All reactions were performed in duplicate. The RNA from peripheral blood of confirmed healthy subjects served as reference sample and was included in each PCR plate, to ensure normalization across experiments. Type I IFN score was calculated as previously described (3, 4). The cut-off for high type I IFN scores among patient peripheral samples was defined as the mean plus 3 SD of the corresponding IFN scores in HC (cut-off for high type I score: 8).

### Statistical Analysis

All statistical analyses were performed using SPSS v.25.0 (IBM, Armonk, NY, U.S.) and GraphPad Prism 9 (GraphPad Software, San Diego, CA, U.S.), with the level of statistical significance being set at 0.05 for univariate and 0.10 for multivariate analysis, respectively. Chi square or Fisher's exact test were performed to compare the frequencies of categorical variables and Mann-Whitney, or *t*-test were employed for detecting significant differences in numerical variables. Spearman's correlation coefficients were calculated to detect correlations between numerical variables. Backward stepwise logistic regression was applied to explore whether hematological abnormalities are independently associated with diverse COVID-19 related outcomes taking into consideration potential confounders such age and sex.

## Results

### Hematological Abnormalities, Demographics, and Clinical Outcomes of Study Participants

Hematological abnormalities as well as demographics, other laboratory features and outcomes in the whole study cohort are displayed in Table 1 and Supplementary Table 2, respectively. The rates of anemia, leukopenia and thrombocytopenia were 28.5, 14.6, and 24.4%, respectively. Among leukocytes, eosinopenia and lymphopenia were the most prominent abnormalities being found in 56.9 and 43.1%, respectively. The mean age of study participants was 62.9 ± 16.6 years, with 68 (55.3%) of the patients being males and 55 (44.7%) females. Regarding disease severity, 6 (4.9%) patients were asymptomatic, 21 (17.1%) patients had mild, 1 (0.8%) moderate, 61 (49.6%) patients severe, and 34 (27.6%) critical disease. One hundred and seven (87%) patients recovered, 13 (10.6%) patients were intubated, and 16 (13%) patients died. Nine out of 13 intubated patients died (69.2%) and four recovered (30.8%).

**Table 1:** Prevalence of hematological abnormalities among the 123 study participants.

**Hematological findings**	**(*n* = 123)**
Hb (g/L) (mean ± SD)	13.3 ± 2.2
WBC (K/μL) (mean ± SD)	6.3 ±2.4
Neutrophils (K/μL) (mean ± SD)	4.6 ± 2.1
Lymphocytes (K/μL) (mean ± SD)	1.2 ± 0.7
Monocytes (K/μL) (mean ± SD)	0.5 ± 0.5
Eosinophils (K/μL) (mean ± SD)	0.4 ± 0.7
PLT (K/Ml)	226 ± 185
Anemia (%)	28.5
Leukopenia (%)	14.6
Neutropenia (%)	2.4
Lymphopenia (%)	43.1
Eosinopenia (%)	56.9
Thrombocytopenia (%)	24.4

*Hb, Hemoglobin; PLT, platelets; WBC, white blood cells*.

### Association of Hematological Abnormalities With Distinct Demographic Characteristics and Disease Outcomes

We next sought to explore whether hematological abnormalities were associated with distinct demographic variables or disease outcomes. As shown in Table 2, patients with baseline anemia were older and more likely to be asymptomatic compared to those without anemia (age: 68.1 ± 17.2 vs. 60.8 ± 16.0, *p* = 0.01; rates of asymptomatic infection: 11.4 vs. 2.3%, *p* = 0.03, respectively). Moreover, compared to those with normal/high lymphocyte or eosinophil counts, patients with lymphopenia and /or eosinopenia at first evaluation were at higher risk for severe/critical disease (88.7 vs. 70% and 90 vs. 62.2%, *p*-values 0.01 and < 0.001, respectively) and mechanical ventilation (17.3 vs. 5.7% and 15.7 vs. 3.8%, *p*-values 0.04 and 0.03, respectively). No significant associations were detected between age or adverse outcomes with decreased WBC, neutrophil or platelet counts. Of note, male sex was associated with the occurrence of thrombocytopenia, with only 20% of patients with thrombocytopenia being females compared to 52.7% with normal/high platelet counts, *p* = 0.002). No association between platelet count with fibrinogen or D-dimer levels was detected (*r* = −0.12, *p* = 0.89 for fibrinogen and *r* = −0.016, *p* = 0.86 for D-dimers, data not shown). In Supplementary Figure 1, the distribution of full blood counts according to severity status is displayed. Following multivariate regression analysis including potential confounders such as age and sex, we could not detect any independent association of hematological parameters with death (data not shown). Regarding severity scores and intubation risk, lymphopenia and eosinopenia but no other hematological abnormalities such as leuκopenia, anemia, thrombocytopenia, or neutropenia were found to be independently associated with intubation risk or severe outcomes. ORs and corresponding 95% CI are presented in Table 3.

**Table 2:** Association between hematological abnormalities with demographic characteristics and COVID-19 related outcomes.

	**Anemia**	**Normal or elevated** **Hb levels**	***p*-value**
Age (mean ± SD)	68.1 ± 17.2	60.8 ± 16.0	0.01
Females (%)	48.6	43.2	0.59
Asymptomatic (%)	11.4	2.3	0.03
High severity scores (%)	80	77.3	0.74
Intubation (%)	11.8	10.2	0.80
Death (%)	17.1	11.4	0.39
	**Leukopenia** **(*****n*** **=** **18)**	**Normal or elevated** **WBC (*****n*** **=** **105)**	
Age (mean ± SD)	62.7 ± 14.5	63.0 ± 17.0	0.76
Females (%)	38.9	45.7	0.59
Asymptomatic (%)	11.1	3.8	0.18
High severity scores (%)	77.8	78.1	0.97
Intubation (%)	16.7	9.6	0.37
Death (%)	5.6	14.3	0.30
	**Lymphopenia** **(*****n*** **=** **53)**	**Normal or elevated lymphocytes (*****n*** **=** **70)**	***p*** **-value**
Age (mean ± SD)	64.8 ± 15.8	61.5 ± 17.2	0.43
Females (%)	35.8	51.4	0.08
Asymptomatic (%)	5.7	4.3	0.72
High severity scores (%)	88.7	70	0.01
Intubation (%)	17.3	5.7	0.04
Death (%)	18.9	8.6	0.09
	**Neutropenia** **(*****n*** **=** **3)**	**Normal or elevated neutrophils (*****n*** **=** **120)**	***p*** **-value**
Age (mean ± SD)	62.0 ± 14.8	62.9 ± 16.7	0.83
Females (%)	66.7	44.2	0.43
Asymptomatic (%)	0	5	0.69
High severity scores (%)	66.7	78.3	0.63
Intubation (%)	33.3	10.1	0.19
Death (%)	0	13.3	0.49
	**Eosinopenia** **(*****n*** **=** **70)**	**Normal or elevated eosinophils (*****n*** **=** **53)**	***p*** **-value**
Age (mean ± SD)	63.7 ± 15.6	61.9 ± 18.0	0.77
Females (%)	47.1	41.5	0.53
Asymptomatic (%)	1.4	9.4	0.04
High severity scores (%)	90	62.2	<0.001
Intubation (%)	15.7	3.8	0.03
Death (%)	15.7	9.4	0.30
	**Thrombocytopenia (*****n*** **=** **30)**	**Normal or elevated** **PLTs (*****n*** **=** **93)**	***p*** **-value**
Age (mean ± SD)	64.9 ± 13.4	62.3 ± 17.6	0.47
Females (%)	20	52.7	0.002
Asymptomatic (%)	6.7	2.3	0.60
High severity scores *n* (%)	90	74.2	0.06
Intubation (%)	13.3	9.8	0.58
Death (%)	10	14	0.57

*Hb, Hemoglobin; PLT, platelets; WBC, white blood cells; High severity scores, severe and critical disease*.

**Table 3:** Independent associations between eosinopenia and lymphopenia with intubation risk and higher severity scores following adjustment for age and sex in a multivariate logistic regression model.

	***p*-value**	**OR 95%(CI)^†^**
**Lymphopenia**		
Intubation risk	0.04	3.9 [1.1–13.8]
Disease severity	0.03	5.3 [1.1–25.2]
**Eosinopenia**		
Intubation risk	0.04	2.90 [1.0–8.1]
Disease severity	≤ 0.0001	6.9 [2.4–19.8]

*Odds ratios (ORs) and 95% confidence intervals (CIs) are displayed*.

† *Adjusted for age and gender*.

### Association of Type I IFN Score and Hematological Abnormalities Among COVID-19 Patients

**Figure 1:**
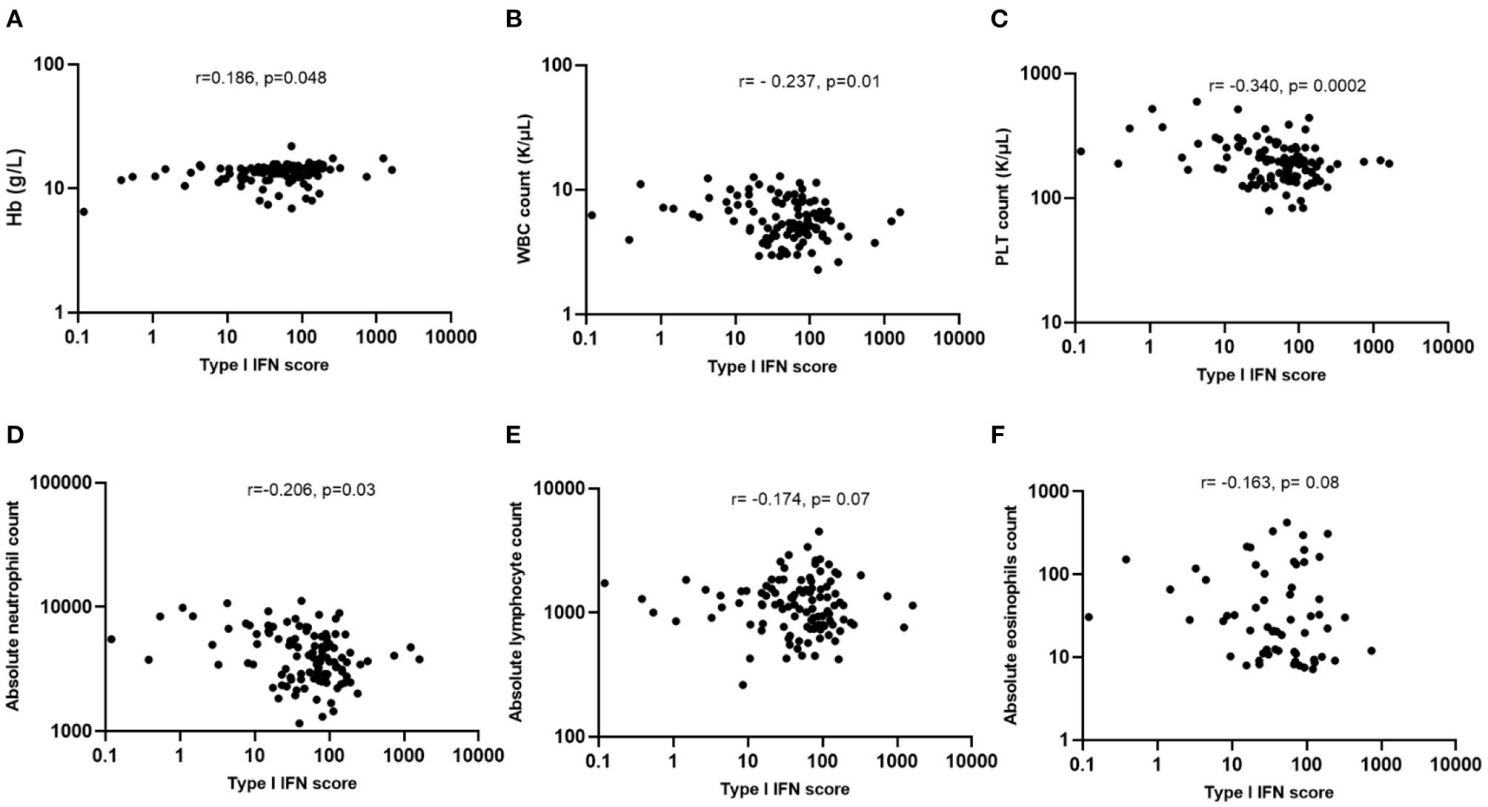
Associations of type I IFN score with hematological features of COVID-19 patients. **(A)** Weak association between type I IFN score and Hb levels (*r* = 0.186, *p* = 0.048); **(B,C)** Statistically significant inverse correlation between type I IFN score with WBC (*r* = −0.237, *p* = 0.01) and PLT counts (*r* = −0.340, *p* = 0.0002); **(D–F)** Statistically significant inverse correlation between type I IFN score with absolute neutrophil (*r* = −0.206, *p* = 0.03), but not with lymphocyte (*r* = −0.174, *p* = 0.07) or eosinophil counts (*r* = −0.163, *p* = 0.08). Hb, hemoglobin; PTL, platelets; WBC, white blood cells; IFN, interferon; *r*, Spearman's correlation.

To identify any possible association of type I IFNs with hematological variables, correlation analysis was performed. As shown in Figure 1, a significantly inverse correlation was found between type I IFN score with WBC (*r* = −0.237, *p* = 0.01, panel B), platelet (*r* = −0.34, *p* = 0.0002, panel C) and absolute neutrophil count (*r* = −0.206, *p* = 0.03, panel D) among COVID-19 infected patients. In contrast, a weak positive correlation was observed between type I IFN score and Hb levels (*r* = 0.186, *p* = 0.048, panel A). No other significant associations between absolute lymphocyte (*r* = −0.174, *p* = 0.07, panel E) or eosinophil (*r* = −0.163, *p* = 0.08, panel F) count with peripheral blood type I IFN score were detected.

**Figure 2:**
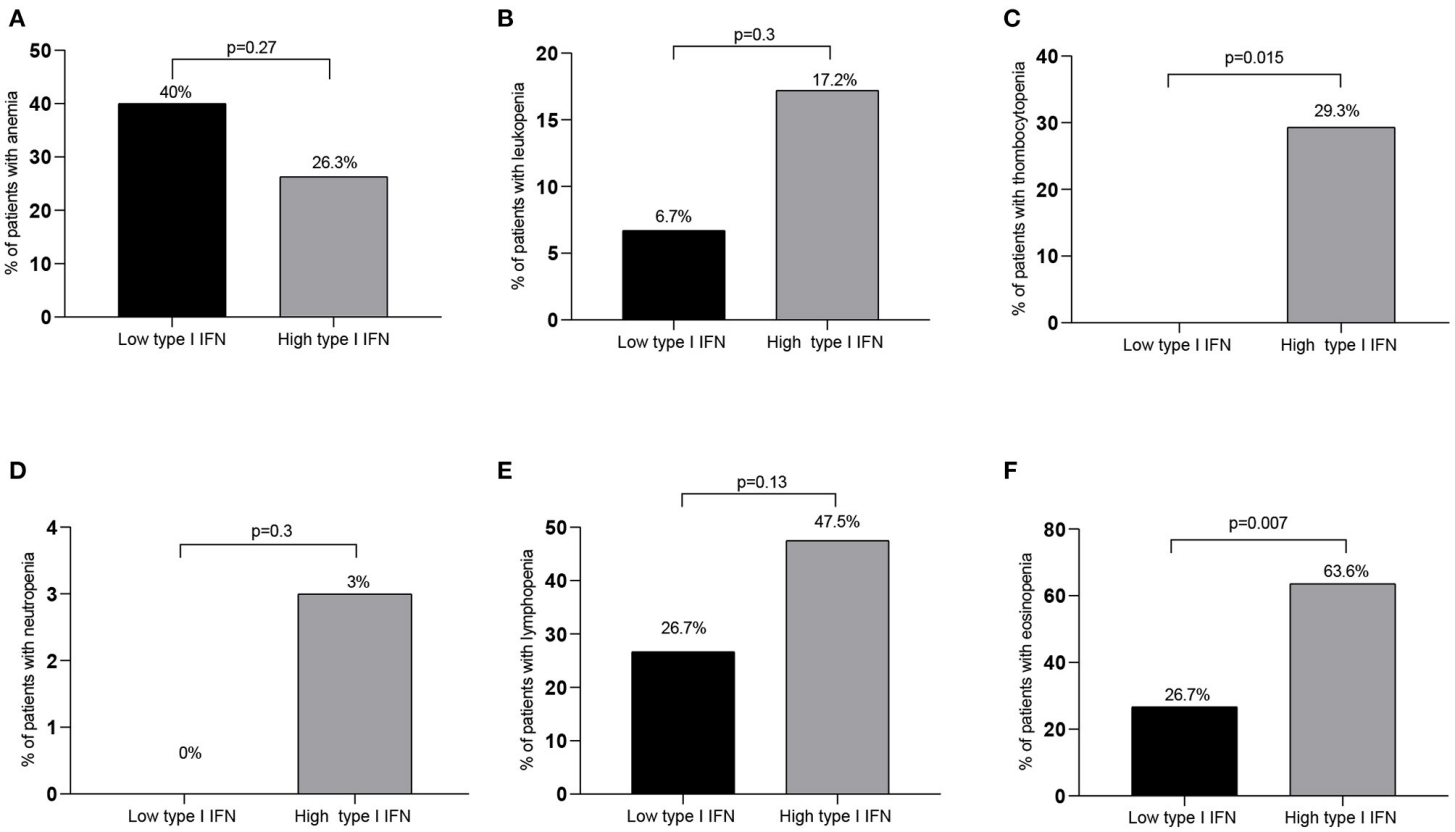
Rates of hematological abnormalities among high vs. low IFN groups. While no significant differences between patient groups with high and low type I IFN peripheral blood scores were detected in the rates of anemia, leukopenia, neutropenia or lymphopenia [**(A,B,D,E)**, respectively], significantly higher rates of thrombocytopenia and eosinopenia were detected in the high compared to the low type I IFN subset **(C,F)**. Only significant differences are shown. IFN, interferon; *p* ≤ 0.05 is considered significant.

In view of the correlations detected between type I IFN score and blood cell counts we next sought to explore whether high type I IFN scores (see Methods) were associated with discrete hematological outcomes. Ninety-nine patients were classified in the high IFN score group and 15 patients were classified in the low IFN group. As displayed in Table 4 and Figures 2C,F, patients displaying high type I IFN score in peripheral blood, had significantly higher rates of thrombocytopenia and eosinopenia compared to those with normal/high platelet or eosinophil numbers (29.3 vs. 0, *p* = 0.015 and 63.6 vs. 26.7%, *p* = 0.007, respectively). No other significant differences in the rates of anemia, leukopenia, neutropenia or lymphocytopenia were detected between high and low type I IFN groups (Figures 2A,B,D,E). No statistically significant differences in outcomes or other laboratory variables were detected between high and low IFN groups (Supplementary Table 3).

**Table 4:** Hematological variables in low and high type I IFN score groups.

	**Low type I** **IFN score** **(*n* = 15)**	**High type I** **IFN score** **(*n* = 99)**	***p*-value**
**Hematological findings**			
Hb (g/L)	12.6 ± 2.5	13.4 ± 2.2	0.33
WBC (K/μL) (mean ± SD)	7.9 ± 2.7	6.1 ± 2.4	0.01
Absolute neutrophil count (mean ± SD)	5,600 ± 2,300	4,500 ± 2,100	0.07
Absolute lymphocyte count (mean ± SD)	1,700 ± 1,100	1,100 ± 500	0.08
Absolute monocyte count (mean ± SD)	500 ± 200	400 ± 500	0.10
Absolute eosinophil count (mean ± SD)	900 ± 1,100	300 ± 700	0.002
PLT (K/Ml)	302 ± 127	216 ± 198	0.001
Anemia (%)	40.0	26.3	0.27
Leukopenia (%)	6.7	17.2	0.30
Neutropenia (%)	0	3	0.49
Lymphopenia (%)	26.7	47.5	0.13
Eosinopenia (%)	26.7	63.6	0.007
Thrombocytopenia (%)	0	29.3	0.015

*CBC, complete blood count; Hb, hemoglobin; IFN, interferon; PTL, platelets; SD, standard deviation; WBC, white blood cells; High severity scores, severe and critical disease*.

## Discussion

To the best of our knowledge, we report for the first time that patients with active SARS-CoV-2 infection with either eosinopenia and/or thrombocytopenia display increased peripheral type I IFN scores compared to their counterparts with normal/high eosinophil and platelet counts. These findings indicate a possible implication of type I IFN activation in hematological alterations commonly observed in COVID 19 patients. While heightened type I IFN scores along with thrombocytopenia were more prominent in male patients, they were not associated with increased risk for severe outcomes including intubation and death or other hematological abnormalities such as anemia, neutropenia or lymphopenia; of note, the latter along with eosinopenia were shown to be associated with more severe outcomes and higher intubation risk, in line with previous observations (10, 11, 17). Of note, patients with normal/high eosinophilic counts but lower hemoglobin levels were more likely to be asymptomatic.

Hematological abnormalities including leukopenia and thrombocytopenia have been previously described as common findings in SARS patients complicated (18) or not (19) by pneumonia. As possible pathogenetic mechanisms, autoimmune destruction, viral or cytokine related insult of hematopoietic stem/progenitor cells, decreased production or increased consumption of platelets in the infected lungs and hypoxia induced mitochondrial disturbances leading to platelet activation and apoptosis, have been all postulated as potential mechanisms (20–23). In the current study, no association between D-dimer levels with platelet counts were detected implying that reduction of platelet numbers is rather independent of local consumption due to thrombotic state (24).

In the setting of SARS-CoV-2 infection, lymphopenia has been previously attributed to direct invasion of lymphocytes and bone marrow by the virus and destruction of the spleen or lymph nodes, to the deleterious effects of cytokine storm on T cells, as well as to elevated lactic acid levels known to reduce lymphocyte proliferation (22). Regarding eosinopenia, the pathophysiology remains ambiguous and multifactorial; inhibition of eosinophilopoiesis, at the level of bone marrow, eosinophilic exhaustion following antiviral enzyme release, and impairment of the interleukin-33 (IL-33) pathway, known to be implicated in eosinophil activation (25) are potential contributory mechanisms. In contrast with lymphocytic infiltration which is identified as a major feature in lung tissue biopsies derived from SARS-CoV-2 patients suffering by acute respiratory distress syndrome, eosinophil recruitment has not been so far reported (26, 27).

The observed association between heightened peripheral blood type I IFN scores with eosinopenia and thrombocytopenia could provide novel insights into underlying mechanisms of cytopenias in the setting of SARS-COV-2 infection. In patients with viral infections and autoimmune diseases a link between type I IFN activation with thrombocytopenia and leukopenia has been previously demonstrated (12, 13) with megakaryocytes expressing functional type I IFN receptors (28). In patients with multiple sclerosis administration of high-dose IFN-β therapy, as disease modifying therapy, has been shown to strongly associate with thrombocytopenia (29) and neutrophil-mediated type I IFN pathway activation in the bone marrow has been shown to affect B cell development in both human and murine lupus (30). Regarding the association of type I IFN and eosinophils, the data are conflicting. Type I IFN has been reported to reduce circulating levels of eosinophil granule proteins, to inhibit eosinophilopoiesis, and negatively regulate production of eosinophil activating cytokines *in vitro* (31). Besides, type I IFN restricts type 2 immune responses, which drive eosinophil recruitment, through the regulation of group 2 innate lymphoid cells (32). However, it should be noted that other studies have shown that type 2 immune responses are mediated by type I IFN in patients with multiple sclerosis and chronic eosinophilic rhinosinusitis (33, 34). The current study, to the best of our knowledge, is the first to directly support a link between type I IFN activation in the setting of in SARS-CoV-2 infection with lower eosinophil and platelet counts, indicating that type I IFN response is a potential mechanism of these hematological alterations observed in COVID-19 disease.

It has been previously suggested that lupus patients with activated type I IFN activity have reduced complement levels (35) and are characterized by impaired endothelial function which in turn associates with increased platelet activation (36). Moreover, impairment of endothelial dysfunction, widespread coagulopathy and complement-induced thrombotic mechanisms have been postulated as major determinants of systemic microangiopathy and thromboembolism among severe cases of COVID-19 (37).

Asymptomatic patients with previous COVID-19 infection have been shown to have downregulated type I IFN inducible genes (38), with SARS-CoV-2 infection delaying the immune system from being activated (39). Recent studies demonstrate that immune cells fail to produce IFN or fail to activate the IFN-mediated response to SARS-CoV-2 infection, rendering the innate immune system unresponsive to viral replication and depriving the cells of the antagonistic action of IFN (40, 41). On the other hand, type I IFN may have an injurious pro-inflammatory effect in severe SARS-CoV-2 infection and therapy with type I IFNs could probably be harmful when administered later in the disease course (42).

Though inborn genetic errors in type I IFN pathway and neutralizing antibodies against type I IFNs leading to impaired type I IFN responses have been associated with adverse outcomes in the setting of SARS-CoV-2 infection (43–45), in the present study we did not detect a direct association between type I IFN peripheral blood score and adverse outcomes such as intubation risk or death. This observation is in accord with a recent meta-analysis by da Silva et al. reporting that plasma protein levels of type I IFN cannot be used as a severity marker for COVID-19 (46). Besides, activation of type I IFN pathway has been previously shown to upregulate the expression of angiotensin converting enzyme-2 receptors in a loop-back mechanism, resulting in further augmentation in intracellular viral load in COVID-19 disease (47).

While previous reports revealed prominent innate immune responses including type I IFN responses among premenopausal women in association with favorable outcomes (48–51), we paradoxically detected an association between type I IFN activation and male gender; this could be confounded by a prominent rate of thrombocytopenia -also shown to be associated with type I IFN responses- among male patients. Besides, premenopausal females are underrepresented in the present study possibly due to less severe disease, not requiring evaluation in a hospital setting. A recent study using artificial intelligence techniques revealed that genes involved in activation of immune pathways (IL-6, TNF, JAK2, IL-1B, SERPINE1, TGFB1, CD8A, and VWF) serve as major hubs in the COVID-19- thrombocytopenia interactomes. Decreased levels of thrombopoietin or its receptors (cMpl) possibly mediated by JAK2 have been postulated as plausible mechanisms (52). Therefore, sex biased immune responses either as a result of distinct sex chromosomes, hormonal exposure or epigenetic variations could partially explain the higher level of inflammation markers and severe cytopenias in SARS-CoV-2 infection observed in males (53, 54).

In view of the above data, JAK inhibitors, already included in the therapeutic armamentarium against COVID-19 (55), hold significant promise in the management of inflammatory and vasculothrombotic manifestations of SARS-CoV-2 infection (47).

Limitations of our single center study are the relatively small number of patients included along with the heterogenicity of disease outcomes. Moreover, beyond type I IFN inducible gene expression, no other proinflammatory cytokines -potentially relevant in COVID-19 related outcomes- were measured.

In conclusion, the current study suggests that hematological abnormalities are commonly detected among SARS-CoV-2 infected patients often in association with severe outcomes and activation of type I IFN pathway. Further studies are needed to confirm these observations.

## Data Availability Statement

The raw data supporting the conclusions of this article will be made available by the authors, without undue reservation.

## Ethics Statement

The studies involving human participants were reviewed and approved by Ethics Committee of Laiko General Hospital (protocol number: 18954-14/12/2020). The patients/participants provided their written informed consent to participate in this study.

## Author Contributions

CM: conceptualization. CM, PL, CS, VG, and NS: methodology. CM, VG, PL, CS, AG, and NS: formal analysis, investigation, and manuscript review and editing. VG and PL: writing—original draft preparation. CM and NS: data and funding acquisition, resources, and supervision. All authors contributed to the article and approved the submitted version.

## Funding

Funding from Molecular Physiology and Clinical Applications Unit, Department of Physiology, National and Kapodistrian University of Athens.

## Conflict of Interest

The authors declare that the research was conducted in the absence of any commercial or financial relationships that could be construed as a potential conflict of interest.

## Publisher's Note

All claims expressed in this article are solely those of the authors and do not necessarily represent those of their affiliated organizations, or those of the publisher, the editors and the reviewers. Any product that may be evaluated in this article, or claim that may be made by its manufacturer, is not guaranteed or endorsed by the publisher.
