# Supplementary material for: Hematological Abnormalities in COVID-19 Disease: Association With Type I Interferon Pathway Activation and Disease Outcomes
Source: Front Med (Lausanne). 2022 Mar 17;9:850472. doi: 10.3389/fmed.2022.850472 (PMC8968418; doi:10.3389/fmed.2022.850472)
Supplement: Supplementary file 1 [file Data_Sheet_1.pdf]

Supplementary Tables

Supplementary Table 1. Specific genes and primers sequence for the gene expression analysi

Primer	Forward Sequence	Reverse Sequence
**GAPDH**	CAACGGATTTGGTCGTATT	GATGGCAACAATATCCACTT
**IFIT1**	CTCCTTGGGTTCGTCTATAAATTG	AGTCAGCAGCCAGTCTCAG
**MX-1**	TACCAGGACTACGAGATTG	TGCCAGGAAGGTCTATTAG
**IFI-44**	CTCGGTGGTTAGCAATTATTCCTC	AGCCCATAGCATTCGTCTCAG

*****GAPDH:glyceraldehyde-3-phosphate dehydrogenase; IFIT1: interferon-induced protein with tetratricopeptide repeats 1; MX-1: myxovirus (influenza virus) resistance 1; IFI44: interferon-induced protein 44

Supplementary Table 2. Demographic, clinical characteristics and laboratory findings of the study population.

**Demographics**	
Age (mean±SD, years)	62.9±16.6
Males n (%)	68 (55.3)
Females n (%)	55 (44.7)
**Disease Severity**	
Asymptomatic n (%)	6 (4.9)
Mild n (%)	21 (17.1)
Moderate n (%)	1 (0.8)
Severe n (%)	61 (49.6)
Critical n (%)	34 (27.6)
**Outcome**	
Recovery n (%)	107 (87)
Death n (%)	16 (13)
Intubation n (%)	13 (10.6)
**Laboratory findings**	
ESR (mm/1 h) (mean±SD)	51 ± 32
CRP (mg/L) (mean±SD)	58.8 ± 67.8
Ferritin (ng/ml) (mean±SD)	654 ±738
Fibrinogen (mg/Dl) (mean±SD)	519 ± 145
Troponin (pg/Ml) (mean±SD)	25.9 ± 38.6
Creatinine (mg/Dl) (mean±SD)	1.5 ± 2.0
Urea (mg/Dl) (mean±SD)	46.6 ± 35.5
SGOT (U/L) (mean±SD)	44.4±76.6
SGPT (U/L) (mean±SD)	43.4±95.6
GGT (U/L) (mean±SD)	46.9±41.9
Alkaline phosphatase (U/L) (mean±SD)	68.5 ± 27.8
CK (U/L) (mean±SD)	226.2 ± 475.0
LDH (U/L) (mean±SD)	307.8 ± 147.0
D-dimers (μg/ml) (mean±SD)	1.45 ± 2.23

*CK: creatine kinase; CRP: C-reactive protein; ESR: erythrocytes sedimentation rate; GGT: gamma-glutamyl transferase; IFN: interferon; LDH: lactate dehydrogenase; SGOT: serum glutamic oxaloacetic transaminase; SGPT: serum glutamic pyruvic transaminase; SD: standard deviation

Supplementary Table 3. Demographics, laboratory findings and outcomes in the low and high type I IFN score groups.

**Laboratory findings**	**Low type I IFN score (n=15)**	**High type I IFN score (n=99)**	**p-value**
**Demographics**			
Age (years) **(**mean±SD)	66.4±19.2	62.0 ±16.3	0.19
Females (%)	11 (73.3)	42 (42.4)	0.03
**Outcomes**			
Asymptomatic %	6.7	5.1	0.79
High severity scores %	80	76..8	0.78
Intubation %	1 (6.7)	11 (11.2)	0.59
Death %	2 (13.3)	12 (12.1)	0.89
**Laboratory findings**			
CRP (mg/L) (mean±SD)	91.7±110.9	52.5±57.3	0.64
Ferritin (ng/ml) (mean±SD)	614.5±506.6	671.4±790.2	0.94
Fibrinogen (mg/dL) (mean±SD)	574.8±254.8	505.5±119.8	0.25
Troponin (pg/mL)	37.8±62.6	25.3±35.1	0.66
Creatinine (mg/dL) (mean±SD)	1.4±1.8	1.6±2.1	0.70
Urea (mg/dL) (mean±SD)	40.1±26.3	48.6±37.4	0.52
SGOT (U/L) (mean±SD)	33.5±22.4	46.6±84.5	0.28
SGPT (U/L) (mean±SD)	30.4±18.3	46.7±106.2	0.94
GGT (U/L) (mean±SD)	50±43	47±43	0.68
Alkaline phosphatase (U/L) (mean±SD)	81±41	66 ±25	0.14
CK (U/L) (mean±SD)	122.6±138.6	212.0±331.3	0.35
LDH (U/L) (mean±SD)	352.9±147.8	301.8±141.9	0.20
D-dimers (μg/ml) (mean±SD)	2.5±4.5	1.3±1.5	0.61

* CK: creatine kinase; CRP: C-reactive protein; ESR: erythrocyte sedimentation rate; GGT: gamma-glutamyl transferase; Hb: hemoglobin; LDH: lactate dehydrogenase; PTL: platelets; SGOT: serum glutamic oxaloacetic transaminase; SGPT: serum glutamic pyruvic transaminase; SD: standard deviation; WBC: white blood cells

Supplementary Figure 1. Cell blood counts according to COVID-19 disease severity status.

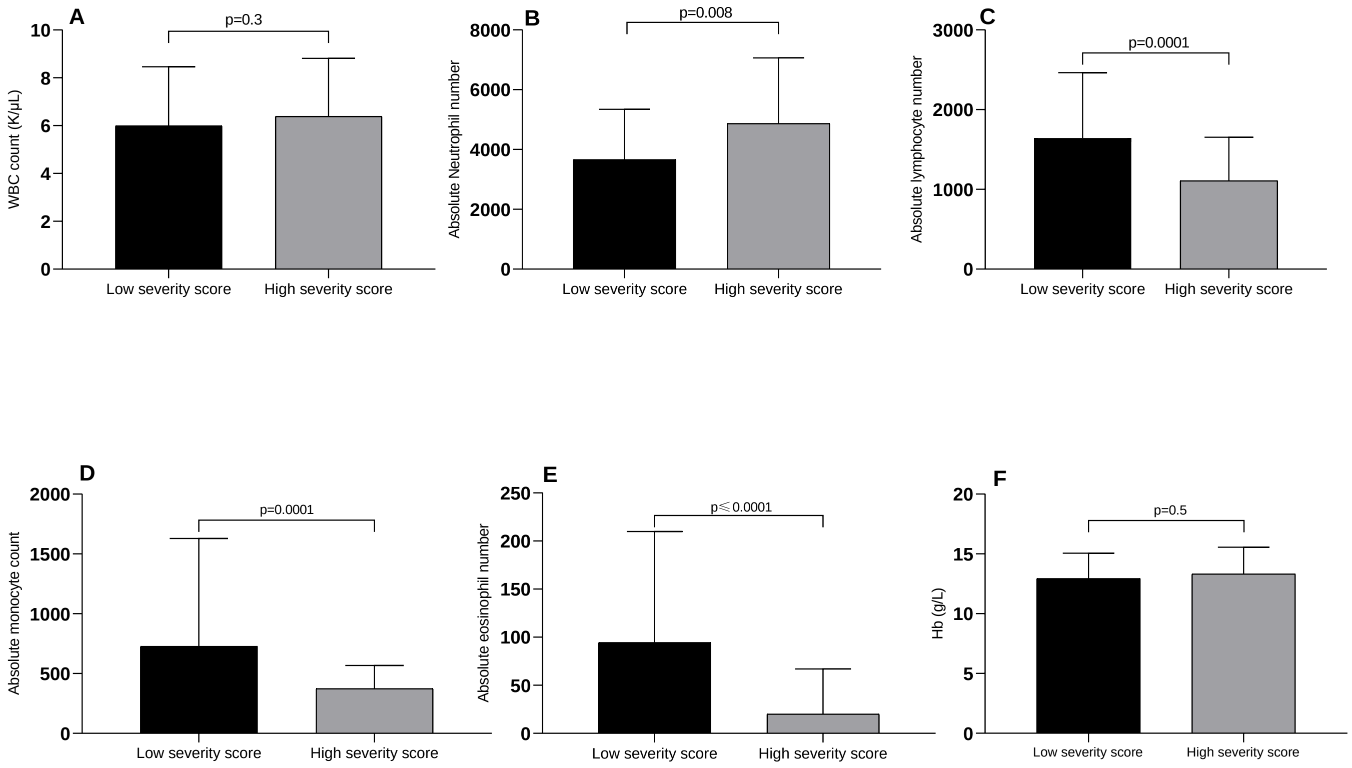

A. WBC number did not differ between COVID -19 patients with low and high severity disease.

B & C. COVID-19 patients with low disease severity display statistically significant lower neutrophil number but higher lymphocyte counts compared to those with high severity disease; D. No difference in PLT number between COVID -19 patients with low and high severity disease score; E. COVID-19 patients with low disease severity display statistically significant higher eosinophil numbers compared to high disease severity subgroup; F. No difference was found in Hb levels between COVID -19 patients with low and high severity disease score.

WBC: white blood cells; Hb: hemoglobin; PLT: platelets; $p \leq 0.05$

In the low severity disease course subgroup have been included and analyzed COVID-19 patients classified according to Center for Disease Control and Prevention (CDC) clinical spectrum of SARS-CoV-2 infection, to asymptomatic, mild and moderate. While in the high severity disease course have been included and analyzed patients with severe and critical illness.
